# Theranostic scope of monometallic selenium and titanium dioxide nanoparticles in biomedicine: A review

**DOI:** 10.1002/hcs2.109

**Published:** 2024-08-13

**Authors:** Shwetha B. Nagarajan, Anuradha Jayaraman, Sanjeevi Ramakrishnan

**Affiliations:** ^1^ Nims Institute of Allied Medical Science and Technology NIMS University Jaipur Rajasthan India

**Keywords:** Monometallic nanoparticles, antimicrobial efficacy, selenium nanoparticles, titanium dioxide nanoparticles, biomedical application, tissue engineering

## Abstract

The nanoparticles (NPs) of metals and metal oxides constitute significant components of technology in terms of monometallic NPs (MNPs). Over the last decade, the most fascinating and in‐depth uses of NPs have been found in the biomedical field, which has demonstrated the therapeutic potential of these particles. Significant strides have been made in the application of nanotechnology across various industries, including biomedical sciences. In biomedicine, two of the most important applications of NPs are in the diagnosis and treatment of disease. Given their ability to deliver specific drugs, these next‐generation NPs provide safe and effective pharmacotherapies for a wide range of disorders. Selenium nanoparticles (SeNPs) and titanium dioxide (TiO_2_) NPs offer potential treatments for various applications, including hair care and cancer treatment. SeNPs help with abiotic stress, plant disease, and growth, while TiO_2_ NPs enhance bio‐imaging and drug delivery. This comprehensive review focuses on MNPs like Se (metal‐based) and TiO_2_ (metal‐oxide based). It covers their synthesis methods, nanoscale physicochemical properties, and the definition of specific industrial applications in various fields of applied nanotechnology, including biomedicine.

## Graphical abstract

The nanoparticles (NPs) of metals and metal oxides constitute significant components of technology in terms of monometallic NPs (MNPs). Throughout the last 10 years, the most fascinating and in‐depth uses of NPs have been found in the biomedical field, which has demonstrated the therapeutic potential of these particles. Significant strides were made in the application of nanotechnology in a variety of industries, including biomedical sciences. In biomedicine, the two most important applications of NPs are in the diagnosis and treatment of disease. Given their ability to deliver specific drugs, these next‐generation NPs provide safe and effective pharmacotherapies for a wide range of disorders. A comprehensive review on MNPs like selenium (metal‐based) and titanium dioxide (metal‐oxide based), their synthesis methods, nanoscale physicochemical properties, and the definition of specific industrial applications in the various fields of applied nanotechnology, including biomedicine, is discussed.

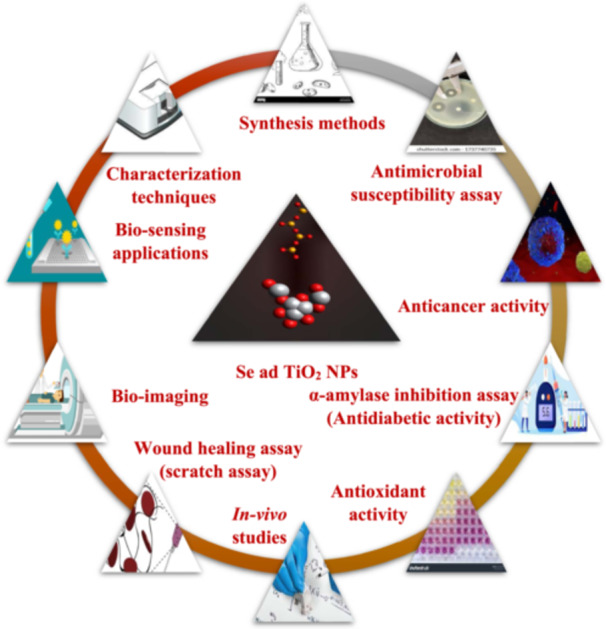

**Abbreviations**
FTIR: Fourier transmission spectroscopy
HR‐TEM: high resolution transmission electron microscopy
MNPs: monometallic nanoparticles
MRI: magnetic resonance imaging
NIR: near‐infrared
NPs: nanoparticles
PSA: particle size analyzer
Se: selenium
SEM: scanning electron microscopy
TEM: transmission electron microscopy
TGA: thermo gravimetric analysis
TiO_2_
: titanium dioxide
XRD: X‐ray diffraction

## 1 INTRODUCTION

Nanoscience is a multidisciplinary technology which incorporates physics, chemistry, biology, medicine, and materials science. Nanoparticles are categorised depending on their origin, dimension, and structure. The initial NPs can be classified as either natural or artificial. Nanoparticles can have zero, one, two, or three dimensions. NPs can have various structures, including liposomes, dendrimers, carbon‐based, or metal‐based. Nanoscale materials, such as SeNPs and TiO_2_ NPs, are gaining recognition as a multidisciplinary study of atoms and molecules on the nanometre scale. Significant improvements are possible with these nanoscale materials in a number of fields, including biology, electronics, energy, biomedicine, environment, pharmacy, and health and medical care. Research on drug development, biomedicine, antimicrobials, and cancer may benefit from the use of SeNPs and TiO_2_ NPs. Their distinct optical and electrical properties allow for their versatile synthesis, which makes them perfect for use in cancer treatment and hair care. The multidisciplinary study of nanoscale materials is critical for their potential applications in a variety of fields.

The present review provide an overview of selenium nanoparticles (metal‐based) and titanium dioxide nanoparticles (metal‐oxide‐based), their synthesis methods, biomedical applications, and how previous studies focused on the green synthesis of Se NPs and TiO_2_ NPs from plants and beneficial microbes, as well as their role in antimicrobial applications, particularly against antibiotic‐resistant bacteria, fungi, and like.

## 2 NANOPARTICLES (NPs)

Nanoscale materials are gaining attention as a sophisticated 21st‐century technology, involving the cross‐disciplinary study of atoms and molecules at the nanometer scale, revealing properties dependent on length scales. Nanoscale materials offer significant scientific and technological advancements in various sectors, including communications, electronics, energy, environment, biology, pharmacy, health care, and medical care. Challenges include miniaturization, smaller volume, lower power consumption, and higher efficiency. From a theoretical standpoint, NPs, characterized by crystalline structures, are intermediate between bulk material and atomic or molecular structures. They can be amorphous or crystalline, transporting liquid droplets or gases. In the 10‐ to 100‐nm‐size range, nanomaterials are crucial for technological advancement, but growth is not always straightforward. NPs, created at the atomic or molecular scale, offer novel physical properties not found in conventional bulk solids. Isodimensional NPs, like spherical silica NPs, have dimensions in the nanoscale range, resulting in enhanced properties [1, 2].

## 3 MONOMETALLIC NPs (MNPs)

As the name implies, MNPs are composed of only one metal. The metal atom determines the properties of these NPs. Depending on the type of metal atom present, MNPs can be magnetic, metallic, or transition metal NPs. They can be produced in a variety of ways, the most common being chemical. Various functional groups can be employed to stabilize their structure. Due to their enhanced physical and chemical properties, metallic NPs have drawn greater attention in recent years. As a result, they are used in a variety of applications such as electrical, optical, and catalysis, among others. Furthermore, they have been employed as antibacterial agents against a variety of pathogens including *E. coli, Streptococcus mutans, Streptococcus pyogens, and Bacillus subtilis* [3]. MNPs exist in various forms, such as pure metal‐based (Se, Au, Ag, Pt, and so on), metal‐oxide based (FeO, Ag_2_O, CuO, MnO_2_, ZnO, Bi_2_O_3_, titanium dioxide (TiO_2_), MgO, Al_2_O_3_, CaO, and so on), metal‐sulfide based, and so on.

### 3.1 Metal‐based NPs

Metal‐based NPs like gold (Au), silver (Ag), selenium (Se), platinum (Pt), and so on. have garnered significant attention in medicine and can currently be manufactured and improved by changing the chemical groups that aid in antibody binding. Noble metal NPs have been employed in a variety of biological applications, including anticancer, radiotherapy enhancement, medication administration, thermal ablation, antibacterial, diagnostic tests, antifungal, and gene delivery. They have several unique features that increase their value. Metal NPs can be functionalized with a range of functional groups, including peptides, antibodies, RNA, and DNA, to target specific cells, as well as potential biocompatible polymers, such as polyethylene glycol. Antimicrobial resistance by pathogenic microorganisms is posing problems for the health care industry [4].

Selenium is an essential element to human health that can be obtained in nature through several sources. In the human body, it is incorporated into selenocysteine, an amino acid used to synthesize several selenoproteins, which have an active center usually dependent on the presence of Selenium. Selenium NPs (SeNPs) are a new metal‐based nanoparticle in the realm of nanotechnology. Nanostructured se increases the surface area available to interact with and kill bacteria in addition to changing the surface morphology to ultimately inhibit the attachment of bacteria. Additionally, SeNPs have shown a sevenfold lower acute toxicity than sodium selenite in mice, showing less prooxidative effects [5].

### 3.2 Metal oxide NPs

Metal oxide NPs have been studied in biomedicine with great success. Such metal oxide NPs with antibacterial activity include Ag_2_O, FeO, MnO_2_, MgO, TiO_2_, and so on. Due to their biocompatibility and capacity to resist bacterial adhesion to titanium‐coated surfaces, titanium, and its alloys are used as important materials in dental implants. TiO_2_ and Ag_2_O NPs are the most well‐studied metal oxide NPs, with excellent antibacterial properties against both gram‐negative and gram‐positive bacteria. Some metal oxides, such as Bi_2_O_3_, ZnO, FeO, MnO_2_, CuO, Ag_2_O, Al_2_O_3_, and others, have antibacterial properties and play important roles in a variety of medical applications. Furthermore, the antibacterial properties and morphology of NPs have been demonstrated. It was discovered that nano‐sized metal particles have a strong bactericidal impact, allowing bacteria to be defeated. The positive charge on the surface of metal‐oxide NPs allows them to adhere to the negatively charged bacteria surface, perhaps increasing the bactericidal action. The form of the nanoparticle is particularly critical since it has a major influence on antibacterial activity. For example, TiO_2_ has been utilized to combat the spread of several infectious diseases. Similarly, metal oxides and doped metal/metal composites such as silver oxide, copper oxide, calcium oxide, magnesium oxide, TiO_2_, and zinc oxide exhibit distinct characteristics, potencies, and spectrum activity, as well as strong antibacterial action. TiO_2_ NPs are nontoxic in nature, inexpensive, biocompatible, reusable, and chemically stable with efficient antimicrobial properties. TiO_2_ NPs are used in many biomedical applications like pharmaceuticals, cosmetics, whiteners, food colorants, and toothpaste (Table 1) [6].

**Table 1:** Characteristic features and applications of metal‐based and metal oxide‐based nanoparticles.

Metal‐based nanoparticles	Metal oxide nanoparticles
Metal‐based nanoparticles are gaining attention in medicine due to their potential for improvement by altering chemical groups that facilitate antibody binding.	Metal oxide nanoparticles, including Ag_2_O, FeO, MnO_2_, MgO, and TiO_2_, exhibit antibacterial activity in biomedicine due to their biocompatibility and resistance to bacterial adhesion on titanium‐coated surfaces.
Noble metal nanoparticles have numerous biological applications, including cancer treatment, radiotherapy, medication administration, thermal ablation, antibacterial, diagnostic tests, antifungal, and gene delivery due to their unique features.	Metal oxide nanoparticles like TiO_2_ and Ag_2_O exhibit antibacterial properties against gram‐negative and gram‐positive bacteria, with others like Bi_2_O_3_, ZnO, FeO, MnO_2_, CuO, Ag_2_O, and Al_2_O_3_ playing significant roles in medical applications.
Metal nanoparticles can be functionalized with various groups like peptides, antibodies, RNA, and DNA to target specific cells, and biocompatible polymers like polyethylene glycol.	Nanoparticles exhibit strong antibacterial properties due to their positive charge and ability to adhere to negatively charged bacteria surfaces, with the form of the nanoparticle significantly influencing its antibacterial activity.
Antimicrobial resistance threatens healthcare, affecting selenium, an essential element for human health. SeNPs is incorporated into selenocysteine, an amino acid used to synthesize selenoproteins.	TiO_2_ and metal oxides and doped metal/metal composites exhibit distinct characteristics, potencies, spectrum activity, and strong antibacterial action, aiding in the prevention of infectious diseases.
SeNPs are a new nanotechnology tool that increases bacteria interaction surface area and inhibits attachment, with a sevenfold lower acute toxicity compared to sodium selenite.	TiO_2_ nanoparticles, nontoxic, inexpensive, biocompatible, and reusable, are used in various biomedical applications like pharmaceuticals, cosmetics, food colorants, and toothpaste due to their efficient antimicrobial properties.

Abbreviations: SeNPs, Selenium nanoparticles; TiO_2,_ titanium dioxide.

The concepts of metal‐based MNPs like selenium and metal‐oxide‐based MNPs such as TiO_2_ are the subject of this review, along with their synthesis techniques, characterization techniques, and biomedical applications.

**Figure 1:**
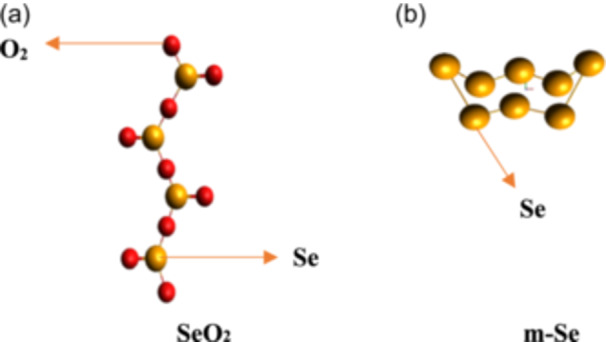
Selenium nanoparticles in (a) SeO_2_ form and in (b) m‐Se form.

**Figure 2:**
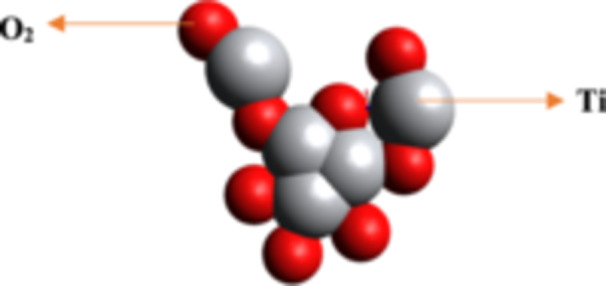
Titanium dioxide (TiO_2_) nanoparticles.

## 4 SELENIUM NPs

Selenium, a crucial component in physics, chemistry, and biology, comes in both inorganic and organic forms. It exists in two crystalline forms: monoclinic and trigonal, the latter being the most stable at room temperature. The synthesis of DNA, thyroid hormone metabolism, immunity to infections and oxidative damage, and reproduction all depend on selenium. It has many industrial and commercial applications due to its excellent catalytic activity in organic hydration and oxidation reactions, high photoconductivity, and low melting point. Selenium is an important trace element in the human body, but the line between usefulness and danger is razor‐thin. It is an important biochemical component of glutathione peroxidase, an enzyme that protects crucial SH‐groups and decomposes peroxides, functioning as an antioxidant. Selenium's bactericidal effect is due to its ability to catalyze the oxidation of intracellular thiols, resulting in the death of microorganism. As a crucial minor component, selenium has been demonstrated to enhance or reinstate the activity of glutathione peroxidase and seleno‐catalysts in preventing free radical damage to cells and tissues in vivo. Selenium is commonly used in dietary supplements and as a potential nutrient in fertilizers. Selenium deficiency can lead to Kashin‐Beck disease, neurological problems, and diseases caused by hemolytic mechanisms. Supplements can prevent viral infections, immune system failure, and neurological problems. SeNPs, with their unique sensing capabilities, have shown promising potential in biological applications as drug carriers, antioxidants, and therapeutic agents, with applications in photocells, photocopying, and renewable energy gadgets (Figure 1) [7].

## 5 TITANIUM DIOXIDE NANOPARTICLES

TiO_2_ NPs, also known as ultrafine or microcrystalline TiO_2_, are widely used in sunscreens due to their high stability, nontoxicity, surface activity, and photocatalytic properties. These NPs have a rutile crystal structure and are considered safer than other UV protection chemicals due to minimal health risks. TiO_2_ NPs, an n‐type semiconductor with a greater band gap, can be enhanced through doping with specific atoms. These NPs, containing Titanium, are widely used in antimicrobial surfaces and have shown potential in enhancing their physical, chemical, and biological properties. Alterations in size and shape can significantly improve their performance. Researchers have studied the effects of TiO_2_ nanostructures like nanoflakes, flowers, and nanotubes in biotechnology. Due to their chemical stability, low toxicity, and multi‐faceted properties, TiO_2_ NPs are increasingly studied for their potential applications in agriculture, nanomedicine, and genetic engineering. TiO_2_ NPs have shown photocatalytic ability and potential for cancer cell destruction and genetic engineering. However, their toxicological profile should be considered (to ensure safe and effective use in various applications) (Figure 2) [8].

## 6 SYNTHESIS OF SELENIUM AND TiO_2_ NPs

SeNPs have been prepared using various approaches, broadly classified into two types: chemical and biological reduction. Biological reduction approach involves the conversion of various organic/inorganic selenium compounds to nontoxic and useful SeNPs using biological agents such as bacteria or extracts of plants. Chemical‐reducing agents are used in chemical reduction (to produce SeNPs) [9]. Researchers have documented microwave, sonochemical, and hydrothermal approaches as methods for reversing chemical reactions, which can be further categorized based on energy source or reaction device. Similarly, based on findings from the cited publication, a number of procedures, including the immediate synthesis method, solvothermal method, sol‐gel method, simple mixing, precipitation method, and microwave‐assisted synthesis may be used to create TiO_2_ NPs [10]. The sol‐gel technique is the process that is utilized to create NPs and nanocomposites. The sol‐gel technique permits the blending of two or more various phases to create hybrids under mild circumstances (Table 2).

**Table 2:** Methods used for SeNPs and TiO_2_ NPs synthesis.

Synthesis of Se nanoparticles	Synthesis of TiO_2_ nanoparticles
I. Chemical reduction method	
The most widely used and straightforward method for synthesizing Se nanoparticles is by reducing Se salts.	TiO_2_ nanoparticles were synthesized using various chemical methods, including solvo‐thermal, sol‐gel, hydrothermal, and co‐precipitation methods.
Sources for oxidation state reduction can be natural substances from plants or microorganisms, as well as reagents and chemicals like ascorbic acid.	The widely used chemical method for synthesizing TiO_2_ nanoparticles has advantages like ease of synthesis and control over NP size and form, but also has limitations.
Chemical reduction involves the use of molecules to reduce elements, salts, or derivatives, with Se nanoparticles being highly sought after for various applications.	This process involves using a closed system (autoclave) at higher temperatures and pressures, utilizing nonaqueous solvents.
II.A. Physical method—Microwave synthesis	
The microwave approach is now a widely used chemical procedure for material production.	Microwave‐assisted methods for synthesizing TiO_2_ are a widely accepted approach.
This synthesis route is a quick, easy, cheap, and clean method that produces a high‐quality end product.	Microwave‐aided procedures are not economically viable due to their high‐power, energy‐expensive nature and the need for homogeneous and rapid heating of reaction mixtures.
Researchers utilized microwave radiation for the production of selenium nanoparticles due to its uniform heat distribution and effectiveness compared to conduction heating.	The microwave‐assisted synthesis method is not feasible for tracking TiO_2_ particle growth over time or manufacturing TiO_2_ nanoparticles in mass quantities.
II.B. Physical method—Hydrothermal synthesis	
The method for preparing biologically acceptable Se NPs is not widely used due to the lack of literature.	The hydrothermal method creates a high‐pressure, high‐temperature reaction environment by using an aqueous solution in a closed vessel, either by heating and pressurizing it.
The approach can produce small particles of 10–20 nm, making it both convenient and environmentally friendly.	Hydrothermal method for preparing TiO_2_ nanoparticles is expensive due to high energy, temperature, and pressure demands, utilizing aqueous solvents.
The hydrothermal technique utilizes wet‐chemical techniques to crystallize materials into nanostructures.	Hydrothermal synthesis of TiO_2_ produces anatase at low temperatures, offering defect‐free nano‐crystals with high specific surface area, good crystallinity, purity, and low energy consumption.
The method of operation is carried out in a sealed container with temperatures between 100°C and 250°C and high vapor pressure. Nanomaterials that are unstable at high temperatures can also be created using the hydrothermal method. Furthermore, it can create high vapor pressure nanomaterials with little material loss.	The method has limitations such as high initial investment, inability to track crystal growth, and its use in supercritical solvent conditions.
Hydrothermal synthesis necessitates expense‐prohibitive autoclaves, posing safety concerns and making it challenging to observe the reaction process.	The expensive equipment, time‐consuming procedure, and maintenance of autoclaves contribute to the high cost and lower yield of TiO_2_ NPs.
III. Green synthesis method	
Over the past two decades, science has shown increased interest in green metal nanoparticle manufacturing, despite the disadvantage of limited cellular absorption.	The chemical processes used to produce TiO_2_ nanoparticles have been found to harm the environment, as the high temperatures and pressure required for synthesis restrict their production.
Se NPs spontaneously produced offer biocompatibility and stability, unlike previous methods that require harmful chemical‐reducing and stabilizing agents, thereby enhancing their applicability in biological systems.	Green nanotechnology is being explored as an environmentally friendly alternative to TiO_2_ NPs production, using biological reducing agents for the preparation of various metallic compounds.
Plant extracts and microorganisms are believed to offer a cost‐effective and safe alternative to chemical procedures in meeting the growing demand for low‐cost manufacturing methods.	Utilizing plants, fruit extract, waste products, and microorganisms in synthesis reduces the use of hazardous and costly chemicals.
Biological extracts act as bio‐reducing agents and nanoparticle stabilizers, with Se NPs biosynthesis simple and requiring no specific equipment or conditions. Examples include bacteria, fungus, protein molecules, algae, and plant extracts.	Green synthesis methods are crucial for large‐scale, low‐cost creation of TiO_2_ NPs.
The biological approach to synthesizing metallic nanoparticles utilizes diverse microorganisms, offering cost‐effectiveness, energy savings, and environmental sustainability over other chemical methods.	Physicochemical methods are expensive, energy‐intensive, and release hazardous chemicals, while biological methods are economical, environmentally safe, reliable, convenient, and simple for synthesizing TiO_2_ NPs.
Biogenic approach to selenium, a crucial element for human and animal health, has advantages like cost, complexity, and potential toxicity, but may enhance efficacy.	The biogenic method for producing TiO_2_ NPs has several drawbacks, including an unfavorable environment, hazardous chemicals, by‐products, and a slow synthesis rate.

Abbreviations: SeNPs, Selenium nanoparticles; TiO2, titanium dioxide.

### 6.1 Chemical reduction method

One common and easy way to produce NPs is through chemical reduction of SeNPs, where the most common sources are natural substances and reagents like ascorbic acid. Chemical reduction uses molecules to reduce an element or its derivatives, so SeNPs are in high demand for a wide range of applications [11]. TiO_2_ NPs are widely used due to their ease of synthesis and control over size and shape. However, these methods have limitations, including cost‐effectiveness, high energy consumption, environmental sustainability, and ecotoxicity. Spray pyrolysis, a low‐cost method for producing TiO_2_ NPs under atmospheric pressure, requires a low temperature, is energy‐intensive, and has limited control over powder properties. Sol‐gel synthesis for TiO_2_ necessitates sol development, gelation, solvent removal, and costly raw materials [12].

### 6.2 Physical reduction methods

#### 6.2.1 Microwave synthesis

The microwave approach is now one of the most common chemical procedures for material production. The approach is quick, easy, cheap, and clean, yields a high yield of the end product, and is commonly referred to as a green synthesis route. Because microwave radiation interacts directly with molecules, it heats more uniformly than conduction heating and is thus more effective. These benefits prompted researchers to use this approach for the production of SeNPs as well [11].

#### 6.2.2 Hydrothermal method

The hydrothermal method is rarely used to produce biologically acceptable SeNPs, but it can produce small particles of 10–20 nm [11]. Hydrothermal and co‐precipitation methods, like TiO_2_ NPs, require elevated temperatures and pressures in autoclaves. Hydrothermal, on the other hand, is expensive, time‐consuming, and yields less due to the high energy, temperature, and pressure requirements. Surfactants and microwave‐assisted methods are widely used in synthesis, but they are energy‐intensive and not economically viable due to high‐power microwave heating [12].

### 6.3 Green synthesis using beneficial microbes

**Figure 3:**
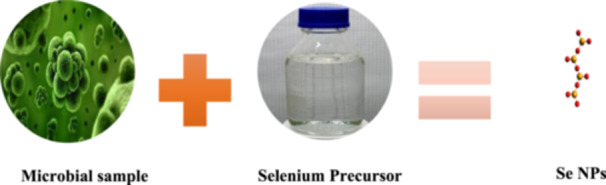
Synthesis of selenium nanoparticles using nonpathogenic microbes.

Bacteria, fungi, and yeasts are commonly used to produce NPs, either intracellular or extracellular. The intracellular method transports ions into the cell, whereas the extracellular method binds or adsorbs metal ions. Sonication, centrifugation, and washing are all steps in the intracellular process. Despite their cost‐effectiveness and environmental compatibility, microorganisms have limitations such as time‐consuming cultivation, difficulty controlling size distribution, polydispersibility, and slower production due to difficulties in selecting the right strain and optimizing reaction parameters. Green metal nanoparticle manufacturing offers advantages over inorganic and organic Se compounds, with biological agents from plant extracts and microorganisms providing a low‐cost, safe alternative to chemical procedures (Figure 3) [13].

Selenium is a micronutrient that everyone needs. SeNPs are gaining popularity as an alternative to the highly toxic selenite. Long et al. [14] looked into the preparation, properties, and cytotoxicity of green synthesized SeNPs using *Paenibacillus motobuensis* LY5201, which was isolated from a local specialty food of longevity area and identified as *P. motobuensis* LY5201. The majority of SeNPs accumulated outside of the cell. SeNPs were roughly spherical, with a diameter of about 100 nm. X‐ray photoelectron spectroscopy and Fourier transform infrared spectroscopy revealed that the purified SeNPs contained both selenium and proteins. Our findings indicated that *P. motobuensis* LY5201 could be an appropriate and robust biocatalyst for SeNPs synthesis. Furthermore, SeNPs' cytotoxicity and anti‐invasive activity on HepG2 were found to be inhibitory, indicating that SeNPs could be used as an anticancer drug (Figure 3).

**Figure 4:**
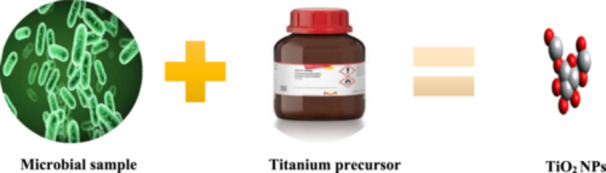
Synthesis of titanium dioxide (TiO_2_) nanoparticles (NPs) using nonpathogenic microbes. Synthesis methodology diagrams of selenium and TiO_2_ NPs are the same only the kind of microbes, metal precursors used varies.

Similarly, bacterial biomass metabolites may aid in the stabilization and bio‐reduction of TiO_2_ NPs. Furthermore, in comparison to bacterial extract, fungal extract has received significant attention due to the benefits of simple extraction, wide‐scale production, economic feasibility, and the enhanced surface of synthesized TiO_2_. As fungi possess enzymes and metabolites which enable them to carry out the reduction of bulk salts into elements of ions, different sizes and shapes of TiO_2_ NPs have been reported, indicating the advantage of green synthesis by microorganisms such as bacteria and fungi. Baker's yeast was used as a cost‐effective approach for the synthesis of anatase small‐sized TiO_2_ NPs with high purity and stability in the context of bacterial TiO_2_ synthesis. Baker's yeast was found to have strong antibacterial action (Figure 4) [15].

Ma et al. [16] found that combining TiO_2_ NPs with *Bacillus mycoides* PM35 improved the morpho‐physio‐biochemical properties of barley (*Hordeum vulgare* L.) under Cd stress. The research investigates the impact of seed priming with different levels of TiO_2_ NPs and soil incubation plant growth‐promoting rhizobacteria (*B. mycoides* PM35) on the biochemical, morphological, and physiological features of Barley plants exposed to different amounts of Cd in the soil. The study found that increasing Cd levels in the soil significantly decreased plant growth and biomass, photosynthetic pigments, gas exchange characteristics, sugars, and nutritional contents from the roots and shoots of the plants. However, the negative impact of Cd toxicity can be overcome by using PGPR and TiO_2_ NPs, which increase plant growth and biomass by capturing reactive oxygen species and decreasing oxidative stress in *H. vulgare*. The results suggest that combining PGPR and TiO_2_ NPs can reduce Cd toxicity in *H. vulgare*, resulting in enhanced plant growth and composition under metal stress.

### 6.4 Green synthesis using plant extracts

**Figure 5:**
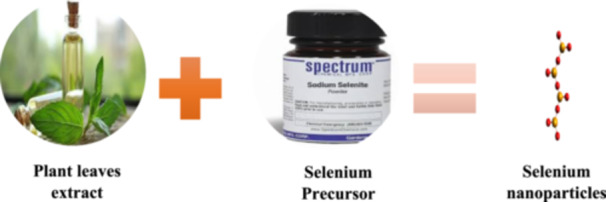
Synthesis of selenium nanoparticles using plant leaves extract.

Scientists are increasingly interested in green metal NPs (SeNPs) manufacturing due to their biocompatibility and stability, but their limited cellular absorption disadvantage makes them less applicable in biological systems compared to traditional methods. Biological agents derived from plant extracts and microorganisms are thought to offer a better alternative to chemical procedures in satisfying the rising demand for low‐cost and safe manufacturing methods. Biological extracts have been found to act as both bireducing agents and nanoparticle stabilizers. There is a wealth of information available on the biosynthesis of SeNPs (Figure 5) [17].

Radhi et al. [18] examined *Staphylococcus aureus* frequency, antimicrobial susceptibility patterns, and risk factors in patients who have burns and infections. This study demonstrates that Se NPs can be utilized to successfully prevent and treat *Staphylococcus aureus* infections and suggested further investigation into such applications. This research synthesized SeNPs using orange peel extract and distilled water. A cross‐sectional study was conducted in Al‐Najaf city hospitals in Iraq, identifying 275 *Staphylococcus aureus* isolates from 630 surgical patients. The isolates were found to be resistant to most antibiotics and capable of forming biofilms and killing host cells. The study found that SeNPs significantly reduced the development of *Staphylococcus aureus* after 24 h at concentrations of 10, 20, 40, and 80 g/mL. The majority of *Staphylococcus* isolates were highly resistant to antimicrobial medications and had the capacity to form biofilms and kill host cells. The study suggests that SeNPs could be used successfully to prevent and treat *Staphylococcus aureus* infections, indicating the need for further research for their potential applications.

A study by Hernández‐Díaz et al. [19] created SeNPs using ascorbic acid (AsAc) as a reducing agent and *Calendula officinalis L*. flowers as a stabilizer. The NPs were tested using various spectrophotometry and transmission electron microscopy (TEM) techniques. Results showed partial antibacterial action and 100% inhibition against *Enterobacter cloacae, Alcaligenes faecalis*, and *Serratia marcescens* bacteria. SeNPs also showed antioxidant activity, highlighting their potential use.

Krishnan et al. [20] used *Dillenia indica* leaf broth to create environmentally friendly green NPs, which were tested for their effectiveness in inhibiting microbiological diseases *Staphylococcus aureus, Serratia marcescens*, and vector mosquitoes *C. quinquefasciatus and A. aegypti*. The NPs were found to damage the peritrophic membrane and disorder epithelial layers, causing histopathological alterations in the larvae. These findings could help develop eco‐friendly chemicals and explain SeNPs' bacterial and mosquito control capabilities.

**Figure 6:**
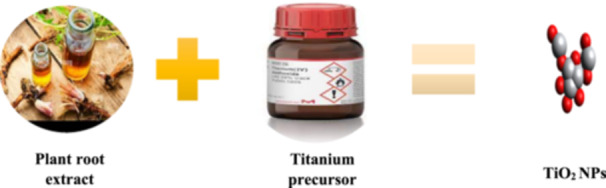
Synthesis of titanium dioxide nanoparticles (TiO_2_ NPs) using plant root extract. Synthesis methodology diagrams of selenium and TiO_2_ NPs are the same only the kind of plant, metal precursors used varies.

Green nanotechnology offers an environmentally friendly alternative to chemical methods for synthesizing TiO_2_ NPs. It uses biological reducing agents and can produce large‐scale, low‐cost TiO_2_ NPs. Plant extract is a popular component due to its safety and feasibility. Plant parts like leaves, roots, and stems can be used for nanoparticle synthesis, with leaves being the most widely used due to high concentration of secondary metabolites (Figure 6) [21].

Experiments on wound healing performed in vivo and histological examinations of treated wounds were utilized to establish the superior wound‐healing effectiveness of TiO_2_ NPs combined with CS gel in diabetic rats. To promote the healing of diabetic wounds, Ahmad et al. [22] produced TiO_2_ NPs utilizing a green synthesis method mediated by *Ocimum sanctum* leaf extract. The UV‐visible spectrum evaluation revealed a high peak between 235 and 320 nm, which was the first proof that TiO_2_ NPs were being biosynthesized. The Fourier transmission spectroscopy (FTIR) measurement was used to qualitatively evaluate the biosynthesized TiO_2_ NPs.

Rajkumari et al. [23] created TiO_2_ NPs using *Aloe barbadensis* aqueous leaf extract. UV‐Vis spectroscopy validated the biosynthesis of TiO_2_ NPs, which were found to be polydispersed, spherical, and 20 nm in size. The preparation of TiO_2_ NPs was confirmed by the presence of strong oxygen and titanium peaks in the EDS pattern. FTIR spectroscopy identified terpenoids, flavonoids, and proteins involved in the production and biosynthesis of TiO_2_ NPs. The TiO_2_ NPs were found to be crystalline and had a strong antibiofilm efficiency against *P. aeruginosa*. The crystalline nature of TiO_2_ NPs in anatase form was confirmed by X‐ray diffraction (XRD) investigation. The TiO_2_ NPs and CS gel demonstrated thixotropic characteristics and pseudoplastic behavior.

## 7 CHARACTERIZATION OF SELENIUM AND TiO_2_ NPs

The performance and properties of biosynthesized NPs can be greatly influenced by their structure, size, and shape, so it is crucial to identify and investigate the physicochemical properties, structure details, purities, and dopants of NPs. The determination of the physicochemical properties of NPs aids in a better understanding of the relationship between these properties and their performance. Due to the presence of numerous macromolecules in their extract that contribute to their own structure, it can be challenging to describe the behavior and structure of green‐synthesized NPs. Common physicochemical characterization techniques for NPs are briefly described in the sections mentioned below [24, 25].

### 7.1 X‐ray diffraction

XRD is a nondestructive analytical technique that can reveal information about a crystalline substance's lattice structure, unit cell dimensions, bond angles, chemical composition, and crystal structure. It is based on the constructive interference of X‐rays and a crystalline sample. XRD is a quick analytical method for determining crystalline material phases. The interaction of X‐rays and the sample results in constructive interference according to Bragg's law, which relates wavelength to diffraction angle and lattice spacing. SeNPs have an XRD range of 10°–90°, while TiO_2_ NPs have an XRD range of 10°–70° with a step of 0.1972°.

### 7.2 Electron microscopy (EM)

For the analysis of NPs, TEM, scanning tunneling microscopy (STM), and scanning EM (SEM) are frequently utilized. An electron gun is used in SEM to generate an electron beam, which is then ejected from the sample. A three‐dimensional image of the sample is produced by gathering scattered electrons and X‐rays. Regarding NP size, shape, localization, dispersity, and aggregation in two‐dimensional images, TEM offers details. Characterizing the NP crystal structure also involves the use of selected area electron diffraction, or SAED.

#### 7.2.1 Scanning electron microscopy

A SEM uses a high‐energy electron beam to scan a sample, revealing its composition, surface topography, and electrical conductivity in high‐resolution images, revealing particles as small as 1–5 nm. SEM micrographs, with their narrow electron beam and large depth of field, provide a unique three‐dimensional view of a sample's surface structure. Bombardment emits electrons, which are captured by a detector, generating a 2D image of the sample surface, which is displayed on a monitor to show spatial variations. Conventional SEMs, with 20X–30000X magnification and 50–100 nm spatial resolution, can scan 1–5 μm widths and analyze specific points for determining sample chemical makeup.

#### 7.2.2 Transmission electron microscope

An electron microscope uses transmission to create diffraction and image images using an objective lens, condenser lenses, and intermediate lenses. The focus on thin samples with low image contrast results in low contrast. The imaging technique involves an objective diaphragm in the rear focal plane to select the transmitted beam and diffracted beam, creating an amplitude‐contrasted image. The bright field (BF) mode distinguishes between crystalline and amorphous portions, while the dark field (DF) mode tilts the incident beam to avoid off‐axis aberrations. SAED and microdiffraction patterns help determine crystal symmetry and interplanar distances, verifying phase identification based on chemical analyses and literature.

### 7.3 Dynamic light scattering (DLS)

This method analyses nanoparticle size and distribution using light interference, Brownian motion, and Strokes‐Einstein equation. The polydispersity index, an autocorrelation function, shows the size distribution range of NPs. Multistage DLS allows for nonspherical NP analysis, also known as Photon correlation spectroscopy (PCS).

### 7.4 Particle size analyzer (PSA)

The PSA technique is ideal for determining the size of NPs. Moreover, samples at much higher concentrations can be measured with backscatter optics thanks to its 90° detection angle, which is not achievable with conventional DLS equipment. When describing NPs, size is an important factor to take into account. The DLS technique is well‐suited to measuring the size of nanoparticle dispersions. Until recently, measuring extremely small, poorly scattering particles or highly diluted samples required the use of powerful lasers.

### 7.5 Ultraviolet‐visible spectroscopy

UV‐visible spectroscopy, also known as UV‐Vis spectrophotometer, is a spectroscopic study of photons in the UV‐Visible region. The visible, UV, and near‐infrared (NIR) light spectrums are used. In this region of the electromagnetic spectrum, there are molecular electronic transitions. The main purpose of UV‐Vis spectrophotometers is to quantify the amount of light that opaque liquids and solids absorb or transmit. It accomplishes this by shining a light beam through the sample and then monitoring the light that remains in a detector. Light with a wavelength between 800 and 200 nm is used by a UV‐Vis spectrophotometer to examine sample electronic transitions. It is difficult to reach a wavelength below 200 nm because oxygen absorbs light at that wavelength. As light travels through the sample, some of the molecules will absorb light at different wavelengths depending on their structural composition and chemical bonding. Electron promotion from HOMO to LUMO results in excited states. Sample molecules absorb light energy, measured by a spectrophotometer.

### 7.6 Thermo gravimetric analysis/differential thermal analyzer (TGA/DTA)

TGA measures a material's weight change in temperature and time, revealing thermal stability and behavior in various environments. DTA measures temperature and heat flow in a material. TGA is a testing method used to identify phase transitions in samples, requiring precise measurements of weight, temperature, and temperature change, and may require transformation of the weight loss curve. Derivative weight loss curves can identify weight loss areas, while thermal gravimetric analysis is used to determine mixture composition and purity by heating the mixture to a temperature high enough for gas decomposition. Stoichiometric ratio (TGA) is a method used in research and testing to calculate the mass percentage of a substance in a sample. It measures the amount of inorganic and organic components, explosive decomposition points, absorbed moisture content, degradation temperatures, and solvent residues. TGA‐DTA/DSC is used to estimate corrosion kinetics in high‐temperature oxidation by measuring a material's weight changes and heat flow based on temperature or time. Techniques like vibrating sample magnetometer, X‐ray photoelectron spectroscopy, Brunauer‐Emmett‐Teller, FTIR, and NMR‐ESR resonance can be used to characterize MNPs, allowing for understanding their size, range, distribution, and structure.

## 8 BIOMEDICAL APPLICATIONS OF SELENIUM AND TiO_2_ NPs

**Figure 7:**
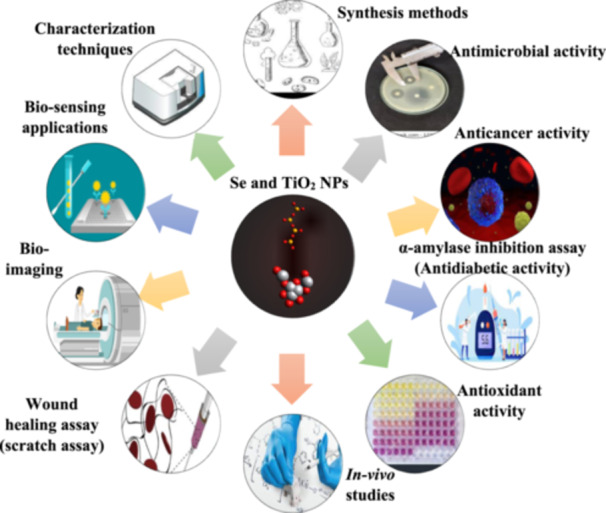
Biomedical applications of selenium nanoparticles (SeNPs) and titanium dioxide (TiO_2_) NPs.

SeNPs are gaining recognition due to their ability to treat diseases such as diabetes, bacterial infections, and cancer. They exhibit anticancer, antibacterial, antioxidant, antidiabetic, and antiparasitic properties. Nano‐antioxidative selenium protects against ROS and free radicals, outperforming traditional medications in these areas. SeNPs can also be used for therapeutic and theranostic applications, overcoming the limitations of chemotherapy, radiation, and other treatments. SeNPs exhibit low toxicity and biomaterial characteristics, making them biologically active and widely available. TiO_2_ NPs, due to their biological and chemical sensitivity, low toxicity, and high chemical stability, have sparked interest for therapeutic applications. Photo‐excited TiO_2_ NPs can produce a nucleic acid endonuclease, potentially killing cancer cells (Figure 7) [26, 27, 28, 29].

### 8.1 Bio imaging

For scientific and biomedical purposes, imaging techniques include spectroscopy methods such as CT scanning, radio imaging using specific nuclides, MRI, and more cutting‐edge scanning techniques like laser ablation, ICPMS, and MALDIMS. Improved diagnostic procedures result in earlier treatment and recovery of patients.

### 8.2 Drug delivery

Traditional forms of medication delivery include oral, parenteral, and nasal routes, which transport the medicine throughout the body. This reduces the drug's efficacy while increasing the risk of side effects. To address these limitations, more efficient medication delivery methods are required. Nanotechnology has been used in biomedicine for targeted drug delivery due to advances in biology, material science, and engineering methodologies.

### 8.3 Phototherapy

Cancer is difficult to treat, which is one of the main reasons why it is one of the most common causes of death worldwide. Surgery is the primary method of treatment, followed by radiation or chemotherapy. Challenges such as drug resistance and drug inaccessibility to tumor cells impact the efficacy of chemotherapy. Phototherapy, like PTT and PDT, has emerged as a novel therapeutic approach for treating a wide range of cancers due to its high effectiveness, low severity, and minimal side effects.

### 8.4 Anticarcinogenic and anticancer activity

Cancers are caused by the death of healthy cells and abnormal tissue growth, with a global increase to 18.1 million cases and deaths in 2018. Treatments include surgery, chemotherapy, and radiotherapy, which target cancer cells and cause cell‐to‐cell death. However, these treatments have limitations, including side effects like anemia and organ damage. New treatments like immune therapy, hyperthermia, and gene therapy have emerged to address these limitations. Although research into the anticancer activity of SeNPs in kidney, lung, breast, and osteosarcoma cancers is ongoing, elemental and naturally occurring selenium cannot be used due to toxicity and bioavailability. Nanoselenium's small size, porosity, and bio‐dispersion make it a beneficial source of selenium, which can prevent diseases and be used in conjunction with radiation and chemotherapy. When administered at the earliest stages of cancer, selenium exhibits strong anticarcinogenic properties. When SeNPs are conjugated with organic moieties and drugs, researchers discovered that they can inhibit cancer growth while increasing anticancer activity and reducing antibiotic toxic effects [29]. The combination of NIR, photothermal, photodynamic, and sonodynamic light therapies with TiO_2_ NPs has demonstrated potential in the treatment of cancer [30].

### 8.5 Antimicrobial activity

**Figure 8:**
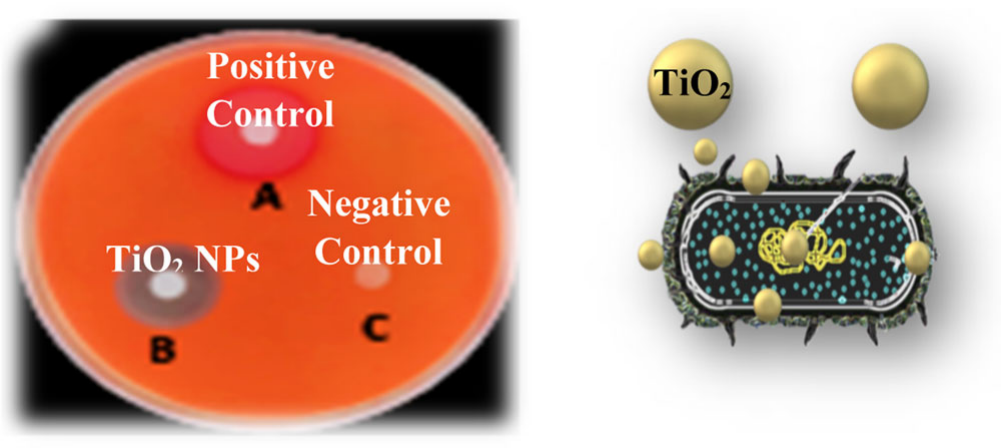
Antimicrobial activity of titanium dioxide nanoparticles (TiO_2_ NPs) and its mechanism.

The stereotypic use of antibiotics results in MDR bacterial strains, posing health and food‐related safety risks. To find novel antibacterial compounds, metal oxide NPs are being studied with an emphasis on preventing bacterial growth. TiO_2_ NPs have been investigated as possible antibacterial agents due to their photocatalytic qualities, chemical stability, nontoxicity, accessibility, and general perception of safety in use. Additionally, because of their antibacterial properties, TiO_2_ NPs are the metal oxide NPs that are being used in biomedicine at a rapid pace. When exposed to UV light, TiO_2_ NPs exhibit photocatalytic antibacterial activity. The antimicrobial effect of TiO_2_ NPs is determined by the thickness of the microbial cell surface, and the order being virus > bacterial wall > bacterial spore. A bacterial cell's membrane experiences oxidative stress when TiO_2_ NPs carry out photocatalysis, which is required for antibacterial activity. This process produces hydroxyl radicals. The photocatalytic activity of TiO_2_ NPs harms the bacterial membrane by promoting the peroxidation of unsaturated phospholipids within the plasma membrane. Significant cellular processes such as oxidative phosphorylation, respiration, and semi‐permeability are also disrupted. Because of thermodynamic and kinetic constraints that hinder inorganic NPs—which are typically hydrophilic—from dispersing in hydrophobic polymer matrices, it is challenging to produce such materials with TiO_2_ NPs. Microbiological infections can cause a wide range of diseases that are extremely harmful to health [31]. Since many antibiotics are still being used, most pathogenic organisms are now resistant to them. The antibacterial properties of TiO2 NPs made by Thakur et al. [32] using leaf extract from *Azadirachta indica* were tested against *Escherichia coli, Bacillus subtilis, Salmonella typhi*, and *Klebsiella pneumoniae*. The study's findings demonstrated that TiO_2_ NPs prevented all of the tested microorganisms from growing. TiO_2_ NPs exhibit a stronger antibacterial effect than TiO_2_ compounds. NPs had the lowest MIC value (10.42 μg/mL) against *Salmonella typhi* and *Escherichia coli*, and the lowest MBC value (83.3 μg/mL) against *Klebsiella pneumoniae* (Figure 8).

**Figure 9:**
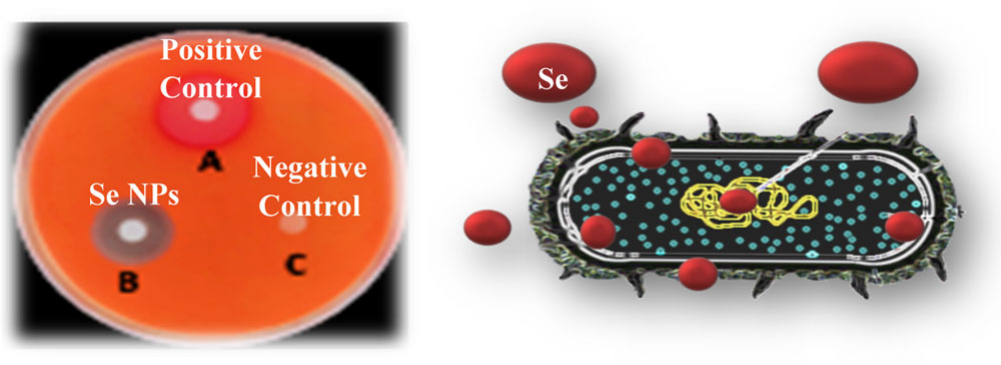
Antimicrobial potential of selenium nanoparticles (SeNPs) and its mechanism.

SeNPs are useful for treating diseases like cancer, diabetes, and inflammatory conditions because they have low toxicity and improved bioavailability. Depending on the intended use, they can be administered intravenously or orally. The development of anti‐infection drugs is being prompted by the increasing resistance of multidrug‐resistant microorganisms and fungi to antimicrobial drugs. Metal oxides, metal NPs, and their mixtures have demonstrated antimicrobial properties. SeNPs, although not significantly reducing biofilm formation, have shown promise in the biomedical industry as an antimicrobial agent for implanted medical devices. Bacterial infections are a global healthcare issue due to antibiotic resistance and side effects from high dosages or prolonged therapy with metalloid and metal‐based NPs [33, 34]. For example, Fardsadegh et al. [35] discovered that Aloe vera leaf extract mediated SeNPs. The study found inhibition zones for both *S. aureus* and *E. coli* bacteria, with *S. aureus* (12 mm) having a larger diameter than *E. coli* (10 mm). Hassan et al. [36] examined the synergistic and individual antimicrobial activities of green synthesized SeNPs and rosemary oil against *Aspergillus fumigatus and Klebsiella pneumoniae*, the most common causes of respiratory disease in cattle. The fungus *A. fumigatus* was detected in 14.28% of nasal swabs, 12% of drinking water, and 32% of animal rations. The bacterium *Klebsiella pneumoniae* was found in 17.4% of tested nasal swabs, 0% in water, and 8% in food. SeNPs showed minimum inhibitory concentrations of 0.4 and 0.5 mg/mL against *A. fumigatus* and *K. pneumoniae*, while rosemary oil had inhibitory concentrations of 0.75 and 1.0 mg/mL, respectively. Synergistic therapy with rosemary oil reduced SeNPs' MIC to 0.1 mg/mL, preventing animal toxicity and breaking down antibiotic resistance (Figure 9).

SeNPs and TiO_2_ NPs are used in studies examining their antidiabetic, antibiofilm, biosensing, and larvicidal properties, as well as in vivo studies (Table 3).

**Table 3:** Potential activities of SeNPs and TiO_2_ NPs.

		Source			Activity studied		
S. No	NPs	Plant source	Microbial source	Characterization	Pathogen studied	Cell/Assay studied	Reference
1.	SeNPs	*Solanum nigrum*	−	UV–Vis spectroscopy, XRD, FTIR, dynamic light scattering, SEM, and Zeta potential	−	Antibacterial, anticancer and anti‐oxidation	Saranya et al. [37]
2.	SeNPs	*Portulaca oleracea*	−	FT‐IR, TEM, SAED, and XRD analyses	*S. aureus B. subtilis E. coli and P. aeruginosa, C. albicans, C. glabrata, C. tropicalis, and C. parapsilosis*	−	Fouda et al. [38]
3.	SeNPs	−	*Bacillus megaterium*	UV‐Vis spectroscopy, DLS, XRD, and TEM	*Rhizoctonia solani*	−	Hashem et al. [39]
4.	SeNPs	*Abelmoschus esculentus*	−	UV‐Vis spectroscopy, XRD, TEM	*S. aureus, S. mutans, E. coli, and P. aeruginosa*	−	Ghaderi et al. [40]
5.	SeNPs	−	*Penicillium corylophilum*.	UV–Vis spectroscopy, TEM, FT‐IR, XRD, EDX, DLS	−	*Anopheles stephensi Wi 38 and Caco‐2 cell lines*	Salem et al. [41]
6.	SeNPs	*Ceropegia bulbosa*	−	UV– Vis spectroscopy, XRD, FT‐IR, HR‐TEM, FE‐SEM‐EDS mapping, zeta potential, and DLS	*B. subtilis and E. coli*	*Aedes albopitus mosquito*	Cittrarasu et al. [42]
7.	SeNPs	*Theobroma cacao*	−	XRD, Z‐potential by DLS, UV‐vis, FT‐IR, and TEM	−	Antioxidant activity	Mellinas et al. [43]
8.	SeNPs	*Withania somnifera*	−	FT‐IR, FE‐SEM, EDX and TEM	*B. subtilis, K. pneumoniae and S. aureus*	−	Alagesan et al. [44]
9.	SeNPs	*Allium sativum*	−	UV‐vis spectrophotometry, SEM, TEM, EDAX, XRD and FTIR	−	Cytotoxicity against Vero cells. CC_50_	Anu et al. [45]
10.	SeNPs	*Clausena dentate*	−	FTIR spectroscopy, UV‐Vis spectroscopy, EDAX, and SEM	−	*Aedes Aegypti, Anopheles stephensi, and Culex quinquefasciatus*	Sowndarya et al. [46]
11.	TiO_2_NPs	*Aloe Vera*	−	XRD, UV–Vis spectroscopy, RAMAN, FTIR, TGA	−	Cytotoxicity to HepG2	Ahmed et al. [47]
12.	TiO_2_NPs	*Azadirachta indica*	−	SEM, XRD, TEM, FTIR, and ultraviolet‐visible spectroscopy	−	Green chemistry‐based benign approach	Shekhar et al. [48]
13.	TiO_2_NPs	*Luffa acutangula*	−	FTIR, XRD, SEM, TEM –SAED and EDX	*B. subtilis, E. coli, S. aureus, E. faecalis, K. pneumoniae and P. aeruginosa fungal strains are A. flavus, A. niger, R. oryzae and S. Rolfsii*	−	Anbumani et al. [49]
14.	TiO_2_NPs	−	*Bacillus spp*.	Uv‐Vis spectrometry, FTIR, Raman spectrometry, XRD, and FESEM	*Candida albicans*	−	Moradpoor et al. [50]
15.	TiO_2_NPs	*Psidium guajava*	−	XRD, FTIR, FESEM and EDX	*S. aureus and E. coli*	Antioxidant activity and phenolic content	Santhoshkumar et al. [51]

Abbreviations: DLS, dynamic light scattering; FTIR, Fourier transmission spectroscopy; SEM, scanning electron microscopy; SeNPs, selenium nanoparticles; TEM, transmission electron microscopy; TiO_2_, titanium dioxide; UV‐Vis, ultraviolet visible; XRD, X‐ray diffraction.

## 9 CONCLUSION

SeNPs and TiO_2_ NPs have potential in biomedicine, antimicrobial, anticancer, and drug development studies. They can be synthesized using various methods, including chemical, hydrothermal, and biogenic methods. Plants and microbes can synthesize these NPs without harming the environment, potentially leading to bio‐masks, hand sanitizers, medicines, and vaccines. SeNPs have intriguing potential in agriculture, food technology, and biomedicine. They can assist with abiotic stress, plant disease, seed germination, and plant growth. In food technology, they can improve nutritional value and control cell damage. They also possess antioxidant and anti‐inflammatory properties and are being used in hair care products like shampoo to provide shine and supplement the hair. Because of to their distinctive optical and electrical characteristics, TiO_2_ NPs, have potential uses in bioimaging and cancer treatment. They can be used as photocatalysts in environmental remediation, nanodrug carriers in targeted drug delivery, and dental and bone implants. The future of nanotechnology in modern medicine is promising.

## AUTHOR CONTRIBUTIONS

**Shwetha B. Nagarajan**: Conceptualization (equal). **Anuradha Jayaraman**: Conceptualization (equal); data curation (equal); visualization (equal); writing—original draft (equal); investigation (equal); writing—original draft (equal); project administration (equal); writing—review and editing (equal). **Sanjeevi Ramakrishnan**: Conceptualization (equal).

## CONFLICT OF INTEREST STATEMENT

The authors declare no conflict of interest.

## ETHICS STATEMENT

Not applicable.

## INFORMED CONSENT

Not applicable.

## Data Availability

Data sharing is not applicable to this article as not data sets are not generated and analyzed during the current study.
